# Supplementary material for: Thin Film Composite Forward Osmosis Membrane with Single-Walled Carbon Nanotubes Interlayer for Alleviating Internal Concentration Polarization
Source: Polymers (Basel). 2020 Jan 23;12(2):260. doi: 10.3390/polym12020260 (PMC7077303; doi:10.3390/polym12020260)
Supplement: Supplementary file 1 [file polymers-12-00260-s001.pdf]

# Thin Film Composite Forward Osmosis Membrane with Single-Walled Carbon Nanotubes Interlayer for Alleviating Internal Concentration Polarization

**Yuanyuan Tang [1a], Shan Li [1a], Jia Xu [1*], Congjie Gao [1]**

[1] Key Laboratory of Marine Chemistry Theory and Technology, Ministry of Education, College of Chemistry and Chemical Engineering, Ocean University of China, Qingdao, Shandong 266100, China

* Correspondence: qdxujia@sina.com.cn.

[a] The authors have equal contributions.

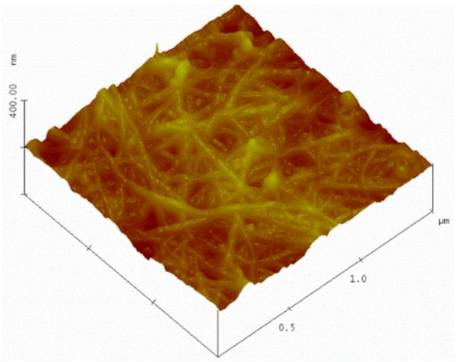


**Figure S1**. Surface AFM morphology of MCE/CNTs10 support layer.

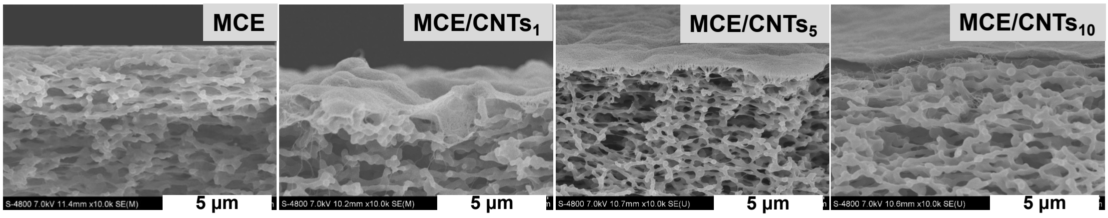


**Figure S2**. Cross-section SEM images of MCE/CNTs support layers.

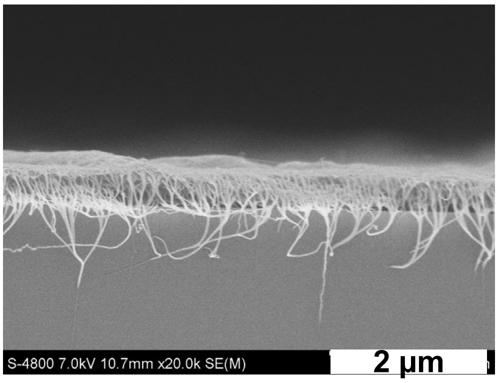


**Figure S3**. Cross-section SEM images of SWCNTs interlayer with the loading of 0.53 g $m^{-2}$.

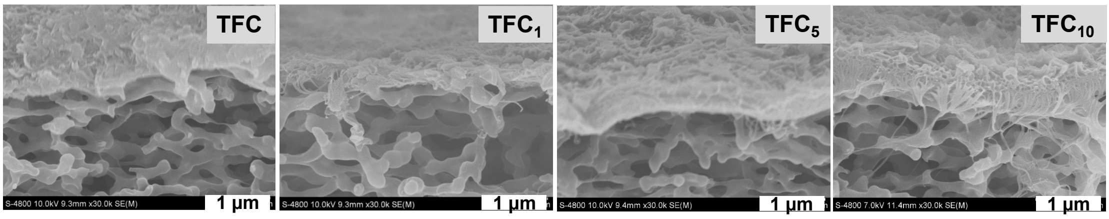


**Figure S4.** Cross-section SEM images of TFC membrane with SWCNTs interlayer.

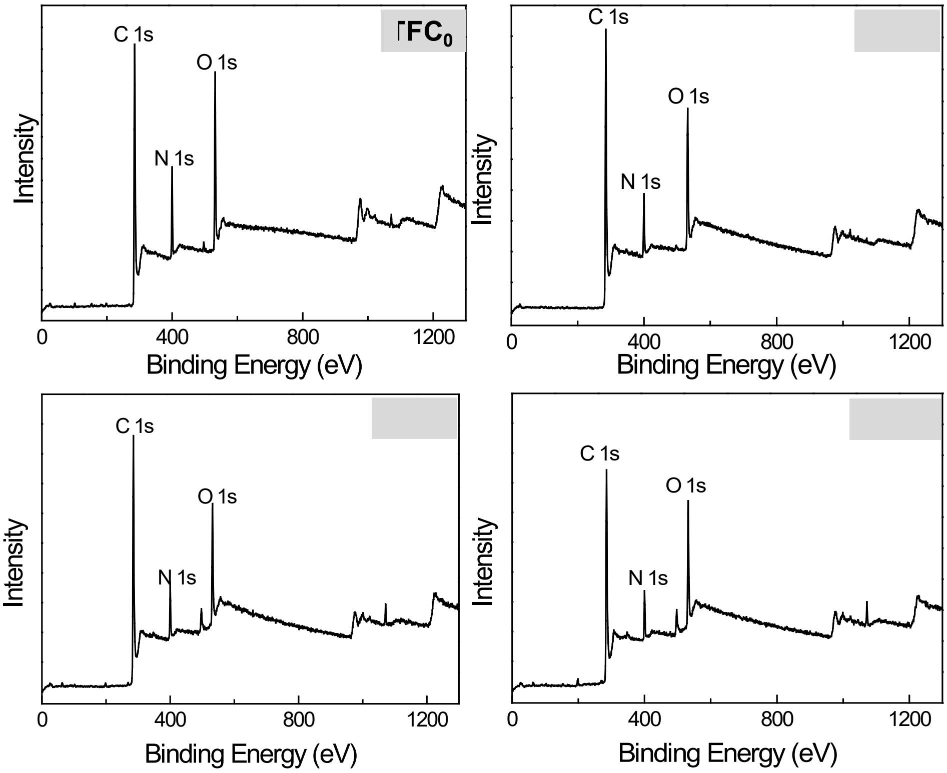


**Figure S5.** XPS survey spectrum of TFC membrane.

**Table S1** Chemical species and composition of TFC membrane analyzed using XPS.

| Membrane | C 1s | | | O 1s | | | N 1s | | |
|---|---|---|---|---|---|---|---|---|---|
| | Energy (eV) | Species | (%) | Energy (eV) | Species | (%) | Energy (eV) | Species | (%) |
| $TFC_0$ | 284.6 | C-C, C-H | 50.5 | 531.5 | N-O=C | 59.1 | 400.0 | N-C=O | 97.7 |
| | 288.0 | N-C=O, O-C=O | 12.1 | 532.8 | O-C=O | 40.9 | 398.5 | $R-NH_2$ | 1.6 |
| | 285.6 | C-N | 37.4 | | | | 401.7 | $R-N^*H_3$ | 0.7 |
| $TFC_1$ | 284.6 | C-C, C-H | 74.7 | 531.5 | N-O=C | 52.6 | 400.0 | N-C=O | 97.0 |
| | 288.0 | N-C=O, O-C=O | 13.3 | 532.8 | O-C=O | 47.4 | 401.7 | $R-NH_2$ | 1.5 |
| | 285.6 | C-N | 12.0 | | | | | $R-N^*H_3$ | 1.5 |
| $TFC_5$ | 284.6 | C-C, C-H | 66.4 | 531.5 | N-O=C | 51.0 | 400.0 | N-C=O | 98.9 |
| | 288.0 | N-C=O, O-C=O | 13.5 | 532.8 | O-C=O | 49.0 | 398.5 | $R-N^*H_3$ | 1.1 |
| | 285.6 | C-N | 20.1 | | | | 401.7 | | |
| $TFC_{10}$ | 284.6 | C-C, C-H | 57.8 | 531.5 | N-O=C | 48.9 | 400.0 | N-C=O | 96.5 |

| | | | | | | | | | |
|---|---|---|---|---|---|---|---|---|---|
| | 288.0 | N-C=O, O-C=O | 20.3 | 532.8 | O-C=O | 51.1 | 401.7 | $R\text{-}N^{*}H_3$ | 3.5 |
| | 285.6 | C-N | 21.9 | | | | | | |
